# How to improve the production of peptidyl compounds in filamentous fungi

**DOI:** 10.3389/ffunb.2022.1085624

**Published:** 2022-12-22

**Authors:** Maiko Umemura, Koichi Tamano

**Affiliations:** ^1^ Bioproduction Research Institute, National Institute of Advanced Industrial Science and Technology (AIST), Tsukuba, Japan; ^2^ Bioproduction Research Institute, National Institute of Advanced Industrial Science and Technology (AIST), Sapporo, Japan; ^3^ Computational Bio Big-Data Open Innovation Laboratory (CBBD-OIL), National Institute of Advanced Industrial Science and Technology (AIST), Tokyo, Japan

**Keywords:** nonribosomal peptide (NRP), ribosomally synthesized and post-translationally modified peptide (RiPP), cyclic peptide, secondary metabolite, heterologous expression

## Abstract

Peptidyl compounds produced by filamentous fungi, which are nonribosomal peptides (NRPs) and ribosomally synthesized and post-translationally modified peptides (RiPPs), are rich sources of bioactive compounds with a wide variety of structures. Some of these peptidyl compounds are useful as pharmaceuticals and pesticides. However, for industrial use, their low production often becomes an obstacle, and various approaches have been challenged to overcome this weakness. In this article, we summarize the successful attempts to increase the production of NRPs and RiPPs in filamentous fungi and present our perspectives on how to improve it further.

## 1 Introduction

Fungal natural products or secondary metabolites are valuable sources for pharmaceutical compounds, such as antibiotics and anticancer drugs, because they have a wide range of structures with unexpected unique bonds and modifications, which are often difficult to chemically synthesize. Penicillin, an antibiotic, and cyclosporin, an immunosuppressant, are examples of clinically used bioactive compounds produced by filamentous fungi. Macrocyclic peptides, such as penicillin, are a major group of fungal natural products, and they are further classified by their biosynthetic pathways into nonribosomal peptides (NRPs), and ribosomally synthesized and post-translationally modified peptides (RiPPs). The former is not synthesized *via* ribosomes, but several amino acid residues are concatenated and cyclized by a large enzyme of thousands or more kilodaltons called nonribosomal peptide synthetase (NRPS), which has domains of adenylation, peptidyl carrier protein, condensation, and thioesterase. On the other hand, the backbone peptides for RiPPs are encoded in their corresponding precursor genes, which are translated by ribosomes in the same manner as ordinary proteins and further modified through such as methylation, prenylation, and cyclization by oxydation.

NRPs and RiPPs are expected to be designed and modified by altering the genes responsible for their synthesis; this aspect makes them more attractive targets for drug discovery. However, there are still many difficulties in making fungal NRPs and RiPPs available in the industry. One of the main issues is their low production, especially in liquid media. It is not unusual that a fungal cyclic peptide cannot be produced in more than 0.1 mg/L yield, even though the corresponding biosynthetic genes are overexpressed. Here, we summarize the current advances in the production of fungal peptidyl compounds and discuss perspectives to overcome their low production based on recent achievements.

## 2 Production of nonribosomal peptides in filamentous fungi

**Figure 1:**
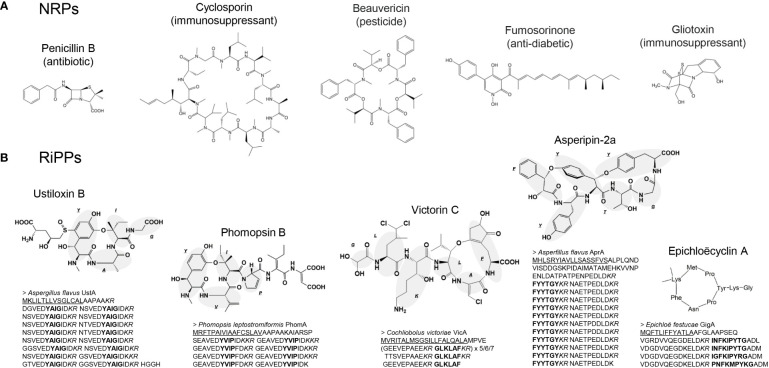
Examples of **(A)** nonribosomal peptides (NRPs) and **(B)** ribosomally synthesized and post-translationally modified peptides (RiPPs) produced by filamentous fungi. In **(A)**, the major known bioactivities are described for each compound. In **(B)**, the precursor protein sequences are shown below the corresponding compounds, where the endoplasmic reticulum signal peptide, core peptides, and Kex2 cleavage sites are underlined, bolded, and italicized, respectively.

Currently, 180 NRPS pathways in Ascomycota are registered in MIBiG (Kautsar et al., 2020), the database of natural compound biosynthetic gene clusters. Within them, there are several valuable NRPs that are used as pharmaceutical agents, such as penicillin and cyclosporin, or that have the potential to be used as such (Keller, 2019) (**Figure 1A**). The production of these NRPs has been improved for industrial use, and random mutagenesis is one of the most frequently applied techniques. For example, penicillin production increased by more than three orders of magnitude from that of the original producer, *Penicillium* spp. wild-type strain, by repetition of random mutagenesis (Fierro et al., 2022). Throughout the repeated random mutagenesis process, both the copy number of penicillin-biosynthetic genes increased, and metabolic reprogramming for more efficient secondary metabolite production occurred (McLean et al., 2015). Specifically, major industrial *Penicillium* strains for penicillin production originated from a naturally-isolated wild-type *Penicillium chrysogenum* strain NRRL1951 (later renamed *Penicillium rubens*) (Houbraken et al., 2011). NRRL1951 was then subjected to repeated mutagenesis, and after 15 generations of mutation, the Wisconsin54-1255 mutant strain with improved penicillin production was developed (Van den Berg, 2011). Wisconsin54-1255 has been used in laboratories for research, and by pharmaceutical companies for development of industrial strains by further random mutagenesis. Thus, most industrial strains are derivatives of Wisconsin54-1255. In addition to mutant development, the liquid fermentation conditions have been optimized. Fed-batch culture was applied aseptically in stainless steel tank reactors of 30,000-100,000-gallon capacity. As a result, 40-50 g/L penicillin yields have been attained to date (Elander, 2003).

However, there are three severe drawbacks to applying random mutagenesis to filamentous fungi: large colony size, slow growth, and the requirement of homokaryon isolation (Tamano and Yoshimi, 2021). Therefore, an approach involving knockout of the targeted gene(s) by recombination and subsequent functional studies, called reverse genetics, is more suitable for improving the production of fungal natural products than random mutagenesis. In this section, we describe what has been accomplished to increase NRP production yield by reverse genetics.

### 2.1 Overexpression of BGC transcription factor and amino-acid-biosynthesis positive regulator genes

A primitive NRP cyclic peptide synthesized by NRPS is usually further modified by other tailoring enzymes as the final NRP. The genes for these enzymes generally accumulate in the chromosome of the producer strain as a biosynthetic gene cluster (BGC) in the same manner as other fungal secondary metabolic genes. A transcription factor gene typically exists within the BGC where it positively regulates the expression of other genes (Keller, 2019). Hence, overexpression of the transcription factor gene by recombination increases the expression of other genes in the BGC, resulting in increase of the production of the corresponding compound. Many NRPs have been reported to be produced at higher yields by overexpressing transcription factor genes (Wang et al., 2021). In the case of the NRP FR901469 produced by an unidentified fungal sp. No. 11243 (Matsui et al., 2015), the production yield was enhanced by repetitive recombination. During the first recombination, the transcription factor gene *frbF* in the BGC was overexpressed, resulting in a 3.4-fold increase in FR901469 production (Matsui et al., 2017a). At the second recombination, another gene, *cpcA*, located outside the cluster, was overexpressed in addition to the *frbF* overexpression. Homologs of CpcA have been reported to positively regulate amino acid biosynthesis in *Neurospora crassa* (Paluh et al., 1988) and *Saccharomyces cerevisiae* (Tian et al., 2007). As a result, FR901469 production further increased by 1.8-fold (Matsui et al., 2017b). Prior to *cpcA* overexpression, Matsui et al. (2017b) found that the addition of some types of amino acids to the culture medium increased FR901469 production. Thus, increased production by *cpcA* overexpression is consistent with previous research.

### 2.2 Overexpression of global regulator genes

Other strategies can be applied in cases of BGCs without transcription factor genes. A global regulatory unit that epigenetically governs gene expression throughout the genome is known to exist in filamentous fungi. It is called the velvet complex, and the LaeA protein in this complex has histone methyltransferase activity. When histones binding to the chromosome are subjected to methylation by LaeA, DNA-histone complexes are dissociated, leading to an increase in the accessibility of RNA polymerases to DNA. As a result, the transcription of the genes located there is facilitated. Some successful cases are reported that genes in the BGC of an NRP were expressed by *laeA* overexpression, increasing the corresponding NRP production up to 25%, 20%, and 126%, respectively, higher than the parental strains (Kosalková et al., 2009; Martín, 2017; Yin et al., 2021).

### 2.3 Increasing copy number of BGC

In addition to modifying gene regulation, increasing gene copy number is also effective. Increasing the copy number of penicillin BGC by recombination caused an increase in penicillin production (Nijland et al., 2010). Interestingly, the industrial strain of *Penicillium rubens* used for mass production, which was bred *via* random mutagenesis, has been proven to have multiple penicillin BGCs (Fierro et al., 1995; Van den Berg, 2011). The same strategy of BGC copy number increase could be applied to heterologous production, which is discussed below.

### 2.4 Optimizing cell physiology and metabolism

Further, optimizing the cell physiology and metabolic pathways involved in NRP biosynthesis is a good approach for improving the production of secondary metabolites. Let us explain using penicillin as an example. Penicillin is synthesized by three enzymes: δ-(l-α-aminoadipyl)-L-cysteinyl-D-valine synthetase (ACVS), isopenicillin N synthase (IPNS), and isopenicillin N acyltransferase (IAT). ACVS is an NRPS and concatenates three amino acids, L-aminoadipic acid, L-valine, and L-cysteine, and then cyclizes the peptide chain. IPNS then forms the β-lactam structure in the intermediate, and IAT confers a phenyl residue to generate the final product. ACVS and IPNS work in the cytosol, whereas IAT works in the peroxisome.

The first successful case to increase penicillin production was the removal of autophagy function from the producer cells. Since autophagosomes possibly degrade enzymes involved in penicillin biosynthesis, the gene *atg1* involved in autophagy was deleted in *P. chrysogenum* (Bartoszewska et al., 2011). Penicillin production increased by 1.4-fold (37%). The second case was the relocation of ACVS to the peroxisome, where the final reaction by IAT was performed. AcvA (a homolog of ACVS) was tagged with peroxisomal targeting signal 1 (PTS1, tripeptide composed of serine, lysine, and leucine) at the C-terminus, increasing penicillin production 3.2-fold (Herr and Fischer, 2014). The third case involved increasing the number of peroxisomes. Overexpression of Pex11-family genes has been reported to increase the number of peroxisomes in mammalian and fungal cells (Marshall et al., 1996; Schrader et al., 1998). With *pex11* overexpression in *Penicillium* sp., the peroxisome number increased, and penicillin production also increased 2-fold (Kiel et al., 2005). The fourth case involved overexpression of the transporter gene. From the transcriptome analysis under penicillin production-facilitating culture conditions, it was found that the *penT* gene encoding a major facilitator superfamily transporter was upregulated, and based on the homology search, it was deduced to be involved in the transport of the penicillin precursor from the cytosol to the peroxisome. Thus, *penT* was overexpressed, and penicillin production increased 2-fold (Yang et al., 2012).

### 2.5 Heterologous production

If it is technically difficult to modify genes in the original producer strains, strains grow very slowly, or protoplasts cannot be sufficiently prepared for transformation, a promising strategy to increase NRP production involves the heterologous expression of the corresponding BGCs in other host strains, where convenient genetic tools are applicable (Alberti et al., 2017; Vassaux et al., 2019). When heterologously expressing a target BGC, it is better to replace the original promoter sequences in the BGC with those of constitutively highly expressed genes in the host strain. This is because the original promoters are related to secondary metabolism, which is often regulated at low levels of expression. Indeed, when the PKS-NRPS metabolite tenellin produced by *Beauveria bassiana* was challenged for heterologous production in *Aspergillus oryzae*, all promoters in the BGC were replaced with those of an *A. oryzae* amylase gene, which is highly expressed in maltose-supplemented media. As a result, a yield of 243 mg/L was attained, which was 5-fold higher than that of the original strain (Heneghan et al., 2010). Enniatin production is another successful example. The gene *esyn1*, which encodes NRPS involved in enniatin biosynthesis in *Fusarium* sp., was heterologously expressed in *Aspergillus niger* using the Tet-on inducible promoter system. This resulted in about 950-fold increase from the initial yield (Richter et al., 2014). Importantly, such BGCs were integrated to chromosomal loci, such as the *pyrG* locus, where genes were highly expressed. High expression in the heterologous host by locus selection can be expected, based on the epigenetic effect of chromosomal gene expression (Palmer and Keller, 2010). In addition, finding such loci at several positions on a chromosome is useful when considering the introduction of several copies of BGC to the heterologous host. Currently, because the BGCs of filamentous fungal NRPs generally reach dozens of kilobases in length, their transfer to other strains requires much labor and a longer period. However, if the original strain is difficult to genetically modify, heterologous production to improve NRP production is a challenge.

## 3 Production of ribosomally synthesized and post-translationally modified peptides in filamentous fungi

Fungal RiPPs were discovered relatively recently. In 2007, amatoxin (a strong food-poisoning toxin with RNA polymerase II inhibitory activity) and phallotoxin (F-actin stabilizer) produced by Amanita mushroom, were the first reported RiPPs in fungi, synthesized through ribosomes (Hallen et al., 2007). In filamentous fungi, ustiloxin B, which has strong inhibitory activity against tubulin assembly (Koiso et al., 1992), was first reported as a RiPP in *Aspergillus flavus* (Umemura et al., 2013; Umemura et al., 2014). Another RiPP class in fungi, later collectively called borosins, found in the basidiomycete *Omphalotus olearius* was reported in 2017 (Ramm et al., 2017). In this article, we focus on RiPPs produced by filamentous fungi or ascomycetes. The precursor protein for ustiloxin B is composed of a signal peptide to the endoplasmic reticulum at its N-terminus, followed by a 16-times repeated sequence that is separated by the recognition site of Kex2 protease into peptides containing the compound backbone (core) peptide. Proteins with these characteristics were later termed Kex2-processed repeat proteins (KEPs) (Marquer et al., 2019). The ustiloxin precursor gene is accompanied by two genes encoding the cyclic factor UstYa homolog (previously called DUF3328 domain-containing protein). RiPPs produced from KEPs as precursor proteins and cyclized by UstYa homolog(s) were named dikaritins (Ding et al., 2016). A bioinformatics survey of 1461 fungal genomes showed that KEPs are conserved among most fungal strains, with at least 838 different types, and 22% of KEP genes are accompanied by UstYa homolog genes (Umemura, 2020). Nevertheless, only five dikaritins, which are the sole examples of RiPPs in filamentous fungi or ascomycetes so far, have been reported: ustiloxin (Umemura et al., 2014), asperipin-2a (Nagano et al., 2016), phomopsin (Ding et al., 2016), epichloëcyclin (Johnson et al., 2015), and victorin (Kessler et al., 2020) (**Figure 1B**). The limited number of reported dikaritins and our experience suggests that most dikaritins are produced under limited conditions or in small quantities. We reported asperipin-2a and its biosynthetic genes from a reverse genetics approach (Nagano et al., 2016; Ye et al., 2019). However, a considerable amount of time and effort was required to obtain a sufficient amount of the compound for identification because asperipin-2a was produced only in a solid maize medium with a < 0.01 mg/kg yield in the original producer strain. The water-soluble properties of dikaritins make them further difficult to prepare because such compounds cannot be concentrated by water-insoluble organic solvents such as ethyl acetate and chloroform; in that sense, asperipin-2a was still better because it could be extracted by *n*-butanol, which is separated from the water layer. Therefore, increasing the production of dikaritins is crucial for obtaining sufficient amounts of compounds to identify absolute structures, and for industrial use.

Similar to NRPs, one of the most direct and effective ways to increase RiPP production is to overexpress the genes responsible for the biosynthesis of target compounds. If there is a transcription factor gene in the target RiPP BGC, it would be better to overexpress it to upregulate the entire BGC. This strategy was used in the case of ustiloxin; the yield of ustiloxin B increased 4.8 times by overexpressing *ustR*, a transcription factor that regulates the whole ustiloxin gene cluster composed of 15 genes (Umemura et al., 2014). In the case of asperipin-2a, we could not apply this strategy because there was no transcription factor gene, at least around the precursor gene. Instead, Ye et al. (2019) succeeded in obtaining 1 mg of asperipin-2a from 400 mL of the potato-maltose liquid medium by heterologous overexpression of the four genes responsible for asperipin-2a biosynthesis originating from *A. flavus*, in *A. oryzae*. The successful heterologous expression of ustiloxin and its intermediates in *A. oryzae* yielded sufficient amounts of compounds to identify their absolute structures (Ye et al., 2016). These two examples show that the heterologous expression strategy should work for the RiPP biosynthetic pathways. When attempting the heterologous production of fungal secondary metabolites, it is important to use a “clean” host, which produces a small number of secondary metabolites by itself, to reduce noisy background signals, observed such as in LC-MS chromatograms. In this regard, *A. oryzae* is one of the best host strains for the heterologous production of fungal natural compounds (Sakai et al., 2012).

Phomopsin (Ding et al., 2016) and victorin (Kessler et al., 2020), both of which are produced by plant pathogenic fungi and are widely known mycotoxins, were identified as RiPPs by deleting the corresponding biosynthetic genes in the producing strains. This was the same for epichloëcyclin (Johnson et al., 2015). Sogahata et al. (2021) demonstrated that three UstYa homologs in the biosynthesis of phomopsin are essential not only for cyclization but also for the desaturation of amino acid moieties; however, they did so not by heterologous expression, but by deleting the corresponding genes in the producing strain. These results and our experiences strongly imply that there are one or more hindrances to the heterologous production of fungal RiPPs, even though the corresponding genes are expressed. Heterologous production of ustiloxin and asperipin-2a, which were originally identified in *A. flavus*, was successful in *A. oryzae*, possibly because *A. flavus* and *A. oryzae* are genetically similar.

Another possible feature of dikaritins that improves their production is the highly repetitive sequence of their precursor proteins. On average, one dikaritin precursor protein contains a 5-fold repeated sequence (Umemura, 2020). We wondered why such highly repetitive sequences are retained ubiquitously in fungi because such structures should be unstable and costly to maintain in the genome. Recently, we reported that the repeat number in a dikaritin precursor protein contributes not only to compound production but also to the transcript level of the precursor protein itself (Umemura et al., 2022). With the increasing number of repeats in the ustiloxin precursor protein, compound production increased quadratically and not linearly. Surprisingly and importantly, the transcript level of the precursor gene increased linearly with an increasing number of repeats. This result suggests that the transcript of the precursor gene is stabilized *via* a highly repeated structure in a feedback manner by an unknown mechanism. As a result, compound production increased quadratically because the factor of repeat number was multiplied by the factor of the linearly increasing transcript level. We do not yet know how the repeated structure of the precursor gene or protein regulates its transcript level. The secondary structure of the highly repeated mRNA may be more stable than that of the unrepeated mRNA, although we could not find any meaningful secondary structure of such mRNAs by computational prediction. The translated protein product might function as a chaperone or RNA-binding protein to protect its transcript. Regardless of the mechanism, the repetitive structure of the ustiloxin precursor protein contributes to compound production, not just as abundant substrates but also as a stabilizer for its transcript. Although this phenomenon has been confirmed only in ustiloxin biosynthesis to date, when attempting to produce sufficient amounts of dikaritins, it might be better to maintain the repetitive structure or even increase the repeat number in dikaritin precursor genes. In addition, overexpression of the precursor protein gene, not the entire biosynthetic pathway, might also increase dikaritin yield.

## 4 Discussion

**Figure 2:**
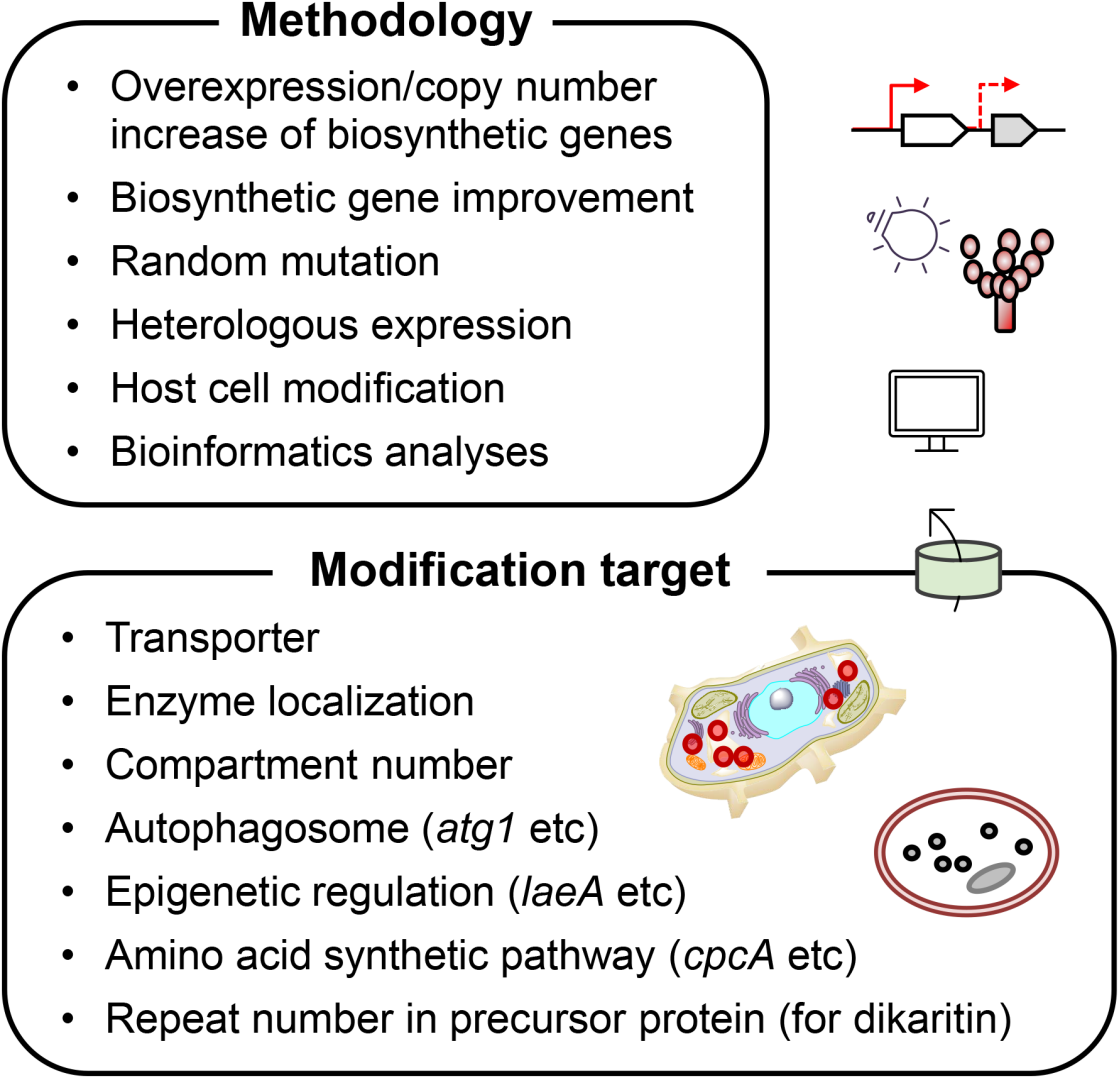
Summary of approaches to improve the production of NRPs and RiPPs in filamentous fungi.

We described successful attempts to increase the production of NRPs and RiPPs in filamentous fungi through reverse genetics and heterologous production (**Figure 2**). Currently, there is still a large hindrance to introducing an entire BGC of NRP or RiPP into other host strains because it is generally large, approximately dozens of kilobases; preparing such a long gene, either by cloning or by artificial gene synthesis, is extremely difficult. Further, it is laborious to express biosynthetic genes for an NRP or RiPP in a heterologous manner when they are located at different loci in the genome, or when some of them are redundant. Genetic techniques to conjugate several DNA fragments instantly, or at least in a few steps, are anticipated to overcome this difficulty.

To modify host strains to improve target compound production, it is reasonable to optimize the localization of the corresponding biosynthetic enzymes in the cells. Some of the fungal peptidyl compounds reviewed by Skellam (2022) are synthesized in compartments. For example, IAT responsible for penicillin biosynthesis, localizes in peroxisomes (Meijer et al., 2010), and SimA functioning as cyclosporin synthetase, localizes in vacuoles (Hoppert et al., 2001). The prolyl oligopeptidase for maturation of amanitin (POPB) may localize in vacuoles (Luo et al., 2010). As mentioned in Section 2, penicillin production was improved using the localization information of biosynthetic enzymes. Probably, for other NRPs and dikaritins, we will be able to improve their production by optimizing the localization of biosynthetic enzymes in cells. To take this approach, the top priority is to elucidate when, where, and how the compounds are synthesized in cells and by which transporter(s) they are transported from one organelle to another; we are currently working on this.

Additionally, in NRPs containing non-proteogenic amino acids such as ornithine (Sheridan et al., 2015), metabolic modification to enhance biosynthesis of non-proteogenic amino acids is likely to increase NRP production. Moreover, for the enzymatic reaction, NRPS requires post-translational modification *via* 4’-phosphopantetheinylation. Therefore, improving 4’-phosphopantetheine biosynthesis by metabolic modification would be useful. Although no successful attempts have been reported to date, this strategy has the potential to be effective.

We have not mentioned the bioinformatics approach in this article, but it is one of the central methodologies for the improvement of compound production. Using bioinformatics, we can narrow down target genes that are bottlenecks for compound production and detect biosynthetic core genes for target NRPs/RiPPs using biological omics data. Indeed, we identified ustiloxin biosynthetic genes using a bioinformatics algorithm, which analyzes genomic information combined with transcriptomic data obtained under compound-producing and non-producing culture conditions (Umemura et al., 2013). In addition to conventional homology search and statistical data analyses of biological large data, machine-learning technologies are rapidly developing in biological fields, which can predict protein structures (Jumper et al., 2021), cell-type-specific gene expression profiles (Newman et al., 2019), protein-protein interactions (Zubek et al., 2015), and points clustered from single-molecule localization microscopy data (Williamson et al., 2020). Using emerging informatics technology, we will be able to take this shortcut to genetically modify filamentous fungal strains to produce target compounds in high yields.

## Data availability statement

The original contributions presented in the study are included in the article/supplementary material. Further inquiries can be directed to the corresponding author.

## Author contributions

All authors listed have made a substantial, direct and intellectual contribution to the work, and approved it for publication.
